# Longitudinal determination of resilience in humans to identify mechanisms of resilience to modern-life stressors: the longitudinal resilience assessment (LORA) study

**DOI:** 10.1007/s00406-020-01159-2

**Published:** 2020-07-18

**Authors:** A. Chmitorz, R. J. Neumann, B. Kollmann, K. F. Ahrens, S. Öhlschläger, N. Goldbach, D. Weichert, A. Schick, B. Lutz, M. M. Plichta, C. J. Fiebach, M. Wessa, R. Kalisch, O. Tüscher, K. Lieb, A. Reif

**Affiliations:** 1 Department of Psychiatry and Psychotherapy, University Medical Center Mainz, Mainz, Germany; 2 Faculty of Social Work, Health Care and Nursing Science, Esslingen University of Applied Sciences, Esslingen am Neckar, Germany; 3 Department of Psychiatry, Psychosomatic Medicine and Psychotherapy, University Hospital Frankfurt, Frankfurt, Germany; 4 Leibniz Institute for Resilience Research (LIR), Wallstraße 7, Mainz, 55122 Deutschland; 5 Neuroimaging Center (NIC), Focus Program Translational Neuroscience (FTN), University Medical Center Mainz, Mainz, Germany; 6 Department of Public Mental Health, Central Institute of Mental Health, Medical Faculty Mannheim, Heidelberg University, Mannheim, Germany; 7 Department of Physiological Chemistry, University Medical Center Mainz, Mainz, Germany; 8 Department of Psychology, Goethe University Frankfurt, Frankfurt am Main, Germany; 9 Brain Imaging Center, Goethe University, Frankfurt, Germany; 10 Department of Clinical Psychology and Neuropsychology, Institute for Psychology, Johannes Gutenberg University Mainz, Mainz, Germany

**Keywords:** Longitudinal, Resilience, Modern-life stressors, Deep phenotyping

## Abstract

Resilience is the maintenance and/or quick recovery of mental health during and after periods of adversity. It is conceptualized to result from a dynamic process of successful adaptation to stressors. Up to now, a large number of resilience factors have been proposed, but the mechanisms underlying resilience are not yet understood. To shed light on the complex and time-varying processes of resilience that lead to a positive long-term outcome in the face of adversity, the Longitudinal Resilience Assessment (LORA) study has been established. In this study, 1191 healthy participants are followed up at 3- and 18-month intervals over a course of 4.5 years at two study centers in Germany. Baseline and 18-month visits entail multimodal phenotyping, including the assessment of mental health status, sociodemographic and lifestyle variables, resilience factors, life history, neuropsychological assessments (of proposed resilience mechanisms), and biomaterials (blood for genetic and epigenetic, stool for microbiome, and hair for cortisol analysis). At 3-monthly online assessments, subjects are monitored for subsequent exposure to stressors as well as mental health measures, which allows for a quantitative assessment of stressor-dependent changes in mental health as the main outcome. Descriptive analyses of mental health, number of stressors including major life events, daily hassles, perceived stress, and the ability to recover from stress are here presented for the baseline sample. The LORA study is unique in its design and will pave the way for a better understanding of resilience mechanisms in humans and for further development of interventions to successfully prevent stress-related disorder.

**Electronic supplementary material:**

The online version of this article (10.1007/s00406-020-01159-2) contains supplementary material, which is available to authorized users.

## Introduction

Recent data from epidemiological surveys in the European Union show that each year, approximately 30% of the population suffer from a mental disorder, such as anxiety, depression, chronic pain, or addiction, that can at least to some extent be traced back to the influence of exogenous or endogenous stressors (e.g., traumatizing events, challenging life circumstances or life transitions, or physical illness) [1]. The high incidence of stress-related disorders, the considerable individual burden, as well as socioeconomic costs associated with them make the promotion of mental health one of the great challenges industrialized countries currently have to face. Progress in our understanding of disease mechanisms and in the development of new therapies in the last decades has been limited despite intense research. The incidence of stress-related mental disorders remains high. It may be, therefore, essential to complement pathophysiological research with an alternative strategy, which is to investigate protective mechanisms that support the maintenance of mental health during and after adversity (e.g., potentially traumatizing events, challenging life circumstances, and physical illness). Focusing on resilience rather than on pathophysiology represents a paradigm shift in mental health research and has great potential for the development of new prevention strategies [2].

Psychological resilience refers to the observation that many individuals do not or only temporarily become ill, despite exposure to significant psychological or physical adversity [3–5]. In this regard, adversity refers to stressors of modern life including ‘macrostressors’ (i.e., potentially traumatizing events, such as man-made or natural disasters) as well as ‘microstressors’ or so-called ‘daily hassles’ (i.e., irritating, frustrating, and distressing demands that to some extent are part of every-day interactions with the environment) [6]. Focusing on mechanisms of resilience, rather than disease, may be a promising approach to promote the prevention of stress-related mental dysfunctions. Resilience has previously been considered to be a stable personality trait [7, 8]. However, nowadays, most theorists define resilience as an outcome, i.e., the absence of mental or related somatic diseases after a potentially traumatizing event, or after a prolonged period of stress [5, 9, 10].

One consequence of this conceptualization of resilience as an outcome is that resilience cannot be measured before an individual has encountered stressors, for example, by cross-sectionally using a personality questionnaire. We recently suggested a conceptual framework for the study of resilience and made proposals for outcome variables (compare [2]). In the simplest possible scenario, we suggested to relate the change in mental health problems (P), measured at two time points (T_A_ and T_B_), to the individual cumulative stressor load (i.e., the sum or amount of stressors) experienced between T_A_ and T_B_. In doing so, one can calculate a parametric score that expresses how an individual’s mental health reacts to stressor exposure. It is assumed that a person is more resilient at T_B_ if that person has developed less mental problems between T_A_ and T_B_ than expected in proportion to the accumulated stressor load. As a consequence, individuals with high cumulative stressor load and low mental health problems at a given time point are considered to be more resilient than, for instance, individuals experiencing an equal stressor load and more mental health problems in that same time period. Necessarily, to operationalize resilience as an outcome in this or comparable ways, prospective, longitudinal study designs are required [9, 11].

Current resilience research is still mainly phenomenological, often restricted to measuring the so-called ‘resilience factors’, that are statistically related to the outcome of resilience. Consequently, published reviews enumerate long lists of resilience factors, which include external factors, such as socioeconomic status or social support, internal factors, such as certain beliefs (e.g., self-efficacy) or skills (e.g., emotion regulation, problem solving), or more recently also neurobiological, (epi)genetic, hormonal, immunological, or other molecular factors [12–15]. Frequently, these factors explain only little variance in the outcome and are difficult to replicate [9]. Moreover, it has been noted that many of these resilience factors overlap conceptually and presumably mediate, correlate with, or depend on each other [2, 15]. For instance, emotion regulation, coping, or problem solving are similar concepts and more distal factors such as social support, life history, or genotype may affect resilience by shaping the way an individual regulates his/her emotions or copes with stressors [2]. This calls for the identification and understanding of mediating mechanisms (‘resilience mechanisms’), i.e., a presumably smaller number of shared cognitive, physiological and/or neural pathways, that provide protection against stress-related impairments.

To identify and investigate such resilience mechanisms, the Collaborative Research Center (CRC) 1193 ‘Neurobiology of resilience to stress-related mental dysfunction: from understanding mechanisms to promoting prevention’, funded by the German Research Foundation (DFG), was established at the Universities of Mainz and Frankfurt and the Leibniz Institute for Resilience Research (LIR; formerly German Resilience Center) in Mainz. The CRC assesses resilience and its underlying mechanisms at several levels of analysis, integrating human studies and animal models in a translational manner. Central to the CRC 1193 is a large longitudinal human cohort study, the Longitudinal Resilience Assessment (LORA) study. This paper aims at providing an overview of the research program, the methods used, and to present baseline data of the LORA study cohort of enrolled healthy subjects.

### Research program

LORA is currently conducted at the University Medical Center Mainz in cooperation with the LIR and the University Hospital of the Goethe University Frankfurt. It comprises a human cohort *N* = 1.191 (Frankfurt: *n* = 611; Mainz: *n* = 580) that has been deep-phenotyped at study entry and is currently followed up for a minimum of 3 years in 18-month intervals. Between baseline assessments, participants are monitored every 3 months for mental health as well as encountered stressors in interim online stressor monitoring during the entire study period.

The two main research aims of the LORA study are:

- *Aim 1* To characterize participants for their resilience to modern-life stressors over time and to operationalize resilience in a quantitative–parametric fashion (i.e., dimensionally rather than categorically).
- *Aim 2* To identify and understand potential underlying mediating mechanisms of resilience to modern every-day life stressors.

Furthermore, LORA has a *third aim* within the CRC 1193 consortium, namely to provide human subprojects within the CRC 1193 with deeply phenotyped subjects, systematically characterized for their longitudinal resilience outcome, for the purpose of experimentally investigating hypothesized resilience mechanisms in these subjects using, among others, neuroimaging and neurobiological methods.

### Tasks

**Fig. 1:**
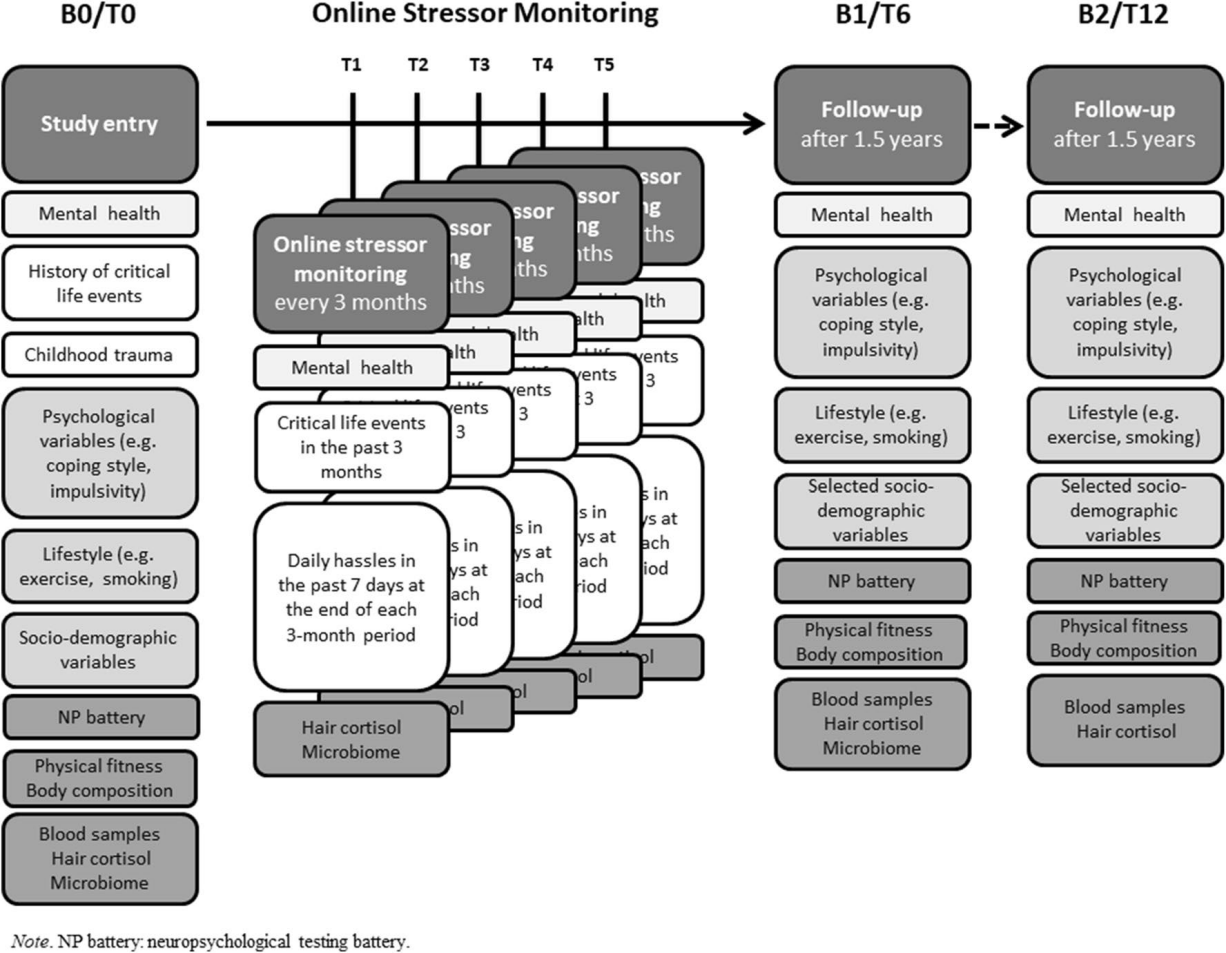
LORA study design and assessment categories

The main tasks to achieve the aforementioned aims are:

1. In-depth phenotyping of *N* = 1.200 subjects at baseline assessments in 18-month intervals, including psychological, sociodemographic, environmental, lifestyle, genetic and epigenetic resilience factors, experienced stress in the months prior to the baseline measurement using hair cortisol samples, and microbiome analyses taken from stool samples in a community sample from the German Rhine-Main region. Also, a neuropsychological test battery is conducted at baseline and every 18 months, investigating emotion regulation, cognitive flexibility, emotional interference inhibition, fear conditioning and extinction, and appraisal styles. Furthermore, repeated quantitative assessments of encountered modern-life stressors, including critical life events and daily hassles, as well as stressor-dependent changes in mental health (via interim online stressor monitoring) and biosamples (hair cortisol and microbiome), are assessed every 3 months to investigate resilience outcomes over time (see Fig. 1). Changes between the baseline assessments, together with information from the interim online assessments, will aid in operationalizing resilience in a dimensional fashion, including possible trajectories of resilience outcomes.
2. Using these data, potential underlying neuropsychological resilience mechanisms and/or biomarkers [such as (epi-)genetic markers and microbiome] shall be identified. Also, these assessments will aid in the identification and understanding of potential underlying mediating mechanisms of resilience to modern every-day life stressors.
3. Subprojects of the CRC 1193 that conduct experimental studies with humans will be provided with subjects that have been systematically characterized for their longitudinal resilience outcome. For this purpose, there are also cooperations with external projects. So far, these include: (1) EU H2020 funded project “Eat2BeNICE”; within the scope of this project, further analyses of the microbiome (via 16S rRNA sequencing) are funded. (2) By means of collaboration with the Psychiatric Genomic Consortium (PGC), we could secure funds for genome-wide genotyping using the PsychChip. (3) A BEDREHELSE project on the epigenetic signature of ADHD allows to perform epigenome-wide analysis of part of the sample using the EPIC array; (4) EU H2020 funded project “DynaMORE” uses amongst others LORA data to develop an in silico model of stress resilience; (5) the State of Rhineland-Palatinate funded the Gutenberg Brain Study (GBS), which is a platform project of the LIR. The GBS has established a population-based sample of 4500 subjects from Mainz. Further collaborations are actively sought for, to leverage the information that is gathered in LORA and interested parties are asked to approach the LORA PIs to this end.

### Description of the LORA study design

The LORA study is a population-based, prospective, longitudinal, multi-center cohort study including adults up to 50 years at study entry. Data collection for the first baseline assessment started on February 1st 2017 and continued until July 15th 2019. Planned longitudinal assessment will be ongoing for at least 3 years.

The study includes baseline assessments at the study centers: at study entry (B0/T0), at 1.5 years (B1/T6) and 3 years (B2/T12), all participants are characterized in detail. Here, phenotyping includes questionnaires on sociodemographic, mental health, life history, psychological, and lifestyle-related variables (including upstream resilience factors) (see Table 1a). Furthermore, biological parameters (blood, stool, and hair samples), anthropometric and current physical fitness components, as well as a neuropsychological test battery are conducted. For the latter, battery tasks can be considered as proxy measures of the putative neurobiological resilience mechanisms (see Table 1b). Between baseline assessments, every 3 months, additional interim online stressor monitoring is conducted during the entire study period (see Fig. 1 for details on study design). The design allows for the assessment of healthy subjects of a large age range and follows them up as they are exposed to naturally occurring life stressors in a modern society.

**Table 1:** (a) Questionnaires and (b) neuropsychological tests used in the LORA study

(a) Questionnaires					
Topic	Questionnaire	*B*	*F*	3m	#*I*
Mental health	General health questionnaire-28 (GHQ-28) [18, 19]	x	x	x	28
	Health questionnaire for patients (PHQ-D) [20, 21]	x	x		16
	Mini international neuropsychiatric interview (M.I.N.I.) [16, 17]	x	x		
*Micro- and macrostressors*					
History of critical life events	Life events checklist from LHC (adapted from Canli et al. [22])	x	x	x	27
Daily hassles	Mainz Inventory of Microstressors (MIMS) [23, 24]	x	x	x	58
Childhood Trauma	Childhood trauma questionnaire (CTQ) [26, 27]	x	x		25
Perceived stress	Perceived stress scale (PSS) [28]; unpublished translation by A. Büssing, University of Witten/Herdecke	x	x	x	10
Maltreatment and abuse	Maltreatment and abuse chronology of exposure (MACE) [29]		x		18
Trauma	Harvard trauma questionnaire (HTQ) [30]		x		35
*Psychological variables*					
Ability to bounce back	Brief resilience scale (BRS) [31, 32]	x	x		6
Cognitive emotion regulation	Cognitive emotion regulation questionnaire (CERQ) [33, 34]	x	x		29
Coping flexibility	Coflex [35]; German version translated by study site	x	x		13
Coping style	Brief Cope [36, 37]	x	x		28
Empathy	Multifaceted empathy test (MET) (subsection: accuracy) [38]	x	x		40
Hardiness	Hardiness Scale ([39]; translated by study site)	x	x		12
Impulsive behavior	Urgency Premeditation Perseverance and Sensation Seeking Impulsive Behavior Scale (UPPS) [40, 41]	x	x		45
Impulsivity	Eight item impulsive behavior scale (I-8) [42]	x	x		8
Interpersonal reactivity	Interpersonal reactivity index (IRI) [43]	x	x		28
Locus of control	Locus of control scale [44, 45]	x	x		28
Optimism	Life orientation test (LOT-R) [46, 47]	x	x		10
Perceived social support	Social support questionnaire (F-SozU) [48]	x	x		14
Personality	Big-five-inventory (BFI-10) [49]	x	x		10
Positive and negative affect	Positive and negative affect schedule (PANAS) [50, 51]	x	x		20
Positive appraisal style	gPASS (Kalisch et al. in prep.)	x	x		29
Resilience factors	Connor–Davidson resilience scale (CD-Risk) [52, 53]	x	x		25
Self-efficacy	General self-efficacy scale (GSE) [54]	x	x		10
Sense of coherence	Orientation to life questionnaire [55, 56]	x	x		29
Social desirability	Social desirability scale-gamma (KSE-G) [57]	x	x		6
State-Trait Anger	State-trait anger anxiety questionnaire (STAXI) [58]	x	x		44
Well-being	WHO questionnaire on well-being (WHO 5) [59, 60]	x	x		5
Identity	Adapted from Skalen zur Messung der ethnischen Identität (MEIM) (GESIS [61])		x		7
Humiliation	Humiliation scale (Lindert and Mollica, in prep.)				26
*Sociodemographic variables*					
General sociodemographic data	General questionnaire for sociodemographic data, family history, ethnical background, employment/salary	x	x		56
Migration	Migration status questionnaire (based on Nesterko and Glaesmer) [62])	x	x		5
Lifestyle variables					
Alcohol use disorder	Alcohol use disorder identification test (AUDIT) [63]	x	x		10
Nicotine dependence	Fagerstrom test for nicotine dependence [64]	x	x		6
Drug consumption	General questionnaire about illegal drug consumption (questionnaire created by study sites)	x	x		6
Physical activity	International physical activity questionnaire (IPAQ) [65, 66]	x	x		27
Physical fitness	International fitness scale (IFIS) [67]	x	x		5
General health variables	General questions concerning health and lifestyle (based on: GESIS [61])				12

Neuropsychological tests					
Resilience mechanism	Task description				
Psychological flexibility	For the assessment of switch costs, stability (distractor inhibition cost), and dispositional flexibility (i.e., the spontaneous switching rate in the face of ambiguous cues); the stability/flexibility task is used. Participants continuously perform a task on digits presented above a fixation cross (ongoing task, e.g., odd/even judgment on digits between 1 and 9). Infrequently, a second digit is presented beneath fixation, and depending on three different brightness conditions, participants have to either switch to the lower digit and perform a different task when the lower digit is brighter (e.g., < / > 5 judgment; flexibility condition) or ignore the lower digit when it is darker than the upper digit (distractor inhibition; stability condition). In the third of three conditions, the brightness difference between the two digits is so subtle that it is not consciously detectable (ambiguous condition). Here, the spontaneous switching rate is examined as an indicator of dispositional cognitive flexibility; established by Armbruster et al. [68]				
Emotional interference inhibition	A classical flanker task, assessing emotional response interference inhibition, during which participants need to respond to a target cue presented in the center of the screen, surrounded by distractor cues. Before each target cue presentation, participants see a picture from the International Affective Picture Set (IAPS) database for 500 ms, differing in emotional valence (i.e., aversive or neutral). Then, a row of seven arrows is presented, which either all point congruently to the left/right side or the target cue points to the opposite site compared to the other arrows (i.e., incongruent trial). Participants are instructed to indicate the pointing direction of the target cue as fast and accurately as possible via a button press on the keyboard with the respective index finger (right index finger for right-pointing target cue; left index finger for left-pointing target cue). Participants receive direct written feedback on the screen for an incorrect or too slow (> 1000 ms) response. After each of five task runs, participants receive feedback about the percentage of correct responses within that run and their average reaction time displayed on the screen. Important outcomes are the reaction time and accuracy differences between congruent and incongruent task trials, between aversive and neutral trial pictures, as well as the interaction of both (congruency x valence); adapted from Stahl et al. [69]				
Positivity bias	Information processing biases favoring positive versus negative information in attention and interpretation will be assessed with a visual probe task (VPT; [70]) and an ambiguous cue task (ACT; a variant of the task paradigm described in Schick et al. 2013 [71], with visual instead of auditory stimulus material), respectively. In the VPT, participants respond to abstract probe stimuli following the presentation of emotional faces (happy, fearful, and neutral). Positivity biases in attention are inferred from accelerated responses to probes that replace happy as opposed to neutral or negative faces. In the ACT, participants learn to associate two visual cues (e.g., a long and a short bar) with positive vs. negative monetary consequences. In the test phase, participants are presented with ambiguous cues (bars of medium length). Responses indicate an individual tendency to interpret ambiguous information as positive or negative				
Volitional emotion regulation	Participants are presented with pictures of differing emotional valence (i.e., aversive and neutral) from the International Affective Picture Set (IAPS). For each picture, participants receive one of three instructions on screen: regard the picture, reappraise (i.e., situation-focused reappraisal), or dissociate. In the regarding condition, participants are expected to carefully look at the picture, take in all its details. In the reappraise condition, participants are asked to change their appraisal of the presented picture scene to regulate their emotions, e.g., by telling themselves that the presented picture is just a scene performed by actors. The dissociation condition is another way of regulating emotions, in which participants are asked to distance themselves actively from the picture content, e.g., by making themselves aware that they do not know the displayed people in the picture. After each picture presentation, participants are asked to rate the intensity of their feeling at that moment as fast as possible on a visual analogue scale from very week to very strong. Participants undergo a short instruction phase with example pictures to get familiar with the different emotion regulation strategies. Main outcomes are differences in the emotion ratings and reaction times and electromyographic (EMG) activity between task conditions; established by Schönfelder et al. [72, 73]. Before the experiment starts, three electrodes are placed on the forehead of the subject for EMG recordings. Two electrodes are placed above the left eyebrow for corrugator muscular activity assessment, while the ground electrode is placed close to the hairline, above all facial muscles (gel-filled electrodes, Biopac^®^ Systems, Inc.)				
Differential fear conditioning (discrimination) and extinction	Classical fear conditioning task, using two different geometric figures as CS + and CS −, respectively, which are counterbalanced either a square or a diamond shape. The CS + is paired with an aversive UCS [i.e., electrodermal stimulation; Digitimer DS7A(CE)] in 50% of all presentations during the acquisition phase by a pain electrode attached to the back of the right hand. Before the acquisition phase, participant’s individual pain threshold is calibrated to reach a pain level that is higher than six on a scale from 0 (“I do not feel anything”) to 10 (“The strongest pain I can imagine being applied with such an electrode”). After each CS + or CS- presentation, participants are asked to rate their level of anxiety, fear or tension as fast as possible on a visual analogue scale from “not at all” to “very much”. During extinction, the CS + is never coupled with the US. After the experiment, participants are asked, which symbol was coupled with the pain stimulation, to make sure that they learned the CS + UCS pairing. During the experiment, participant’s skin conductance rate (SCR) is measured with two electrodes attached to the palm of the left hand (Biopac^®^ Systems, Inc.)				

Notes: *B* baseline, *F* follow-up at main assessments every 18 months; 3m = interim analyses every 3 months; #*I* = number of total items

### Sample recruitment

Participants were recruited via online or printed advertisements, public advertisements (at the local universities, the university medical centers, libraries, shops, and gyms) and a webpage setup specifically for the project (https://lora-studie.de/). Potential participants contacted the study centers via phone or e-mail.

Interested participants were then re-contacted by trained student assistants via phone or letter and provided with study information. They were screened for study eligibility by trained staff using structured in-house developed telephone interviews. Inclusion criteria were: age 18–50 years (the upper age limit is set to minimize potential impacts of organic brain disorders), normal or corrected vision, sufficient mastery of the German language, and the ability to provide informed consent. Sufficient knowledge of German can be inferred from the telephone screening, which takes about 15 min. Also, participants are asked during that screening, whether German is their mother tongue. However, being a non-native German speaker does not lead to study exclusion, as long as the language proficiency is sufficient to understand the content of the phone call. Exclusion criteria were: lifetime diagnosis of schizophrenia or bipolar disorder, organic mental disorders, substance dependence syndromes other than nicotine, as well as any other current severe axis I disorder or current severe medical conditions. Participants with known learning disabilities, serious neurological disorders (e.g., tumors in the central nervous system), or participants who had taken part in a drug trial in the previous 6 months were also excluded.

**Fig. 2:**
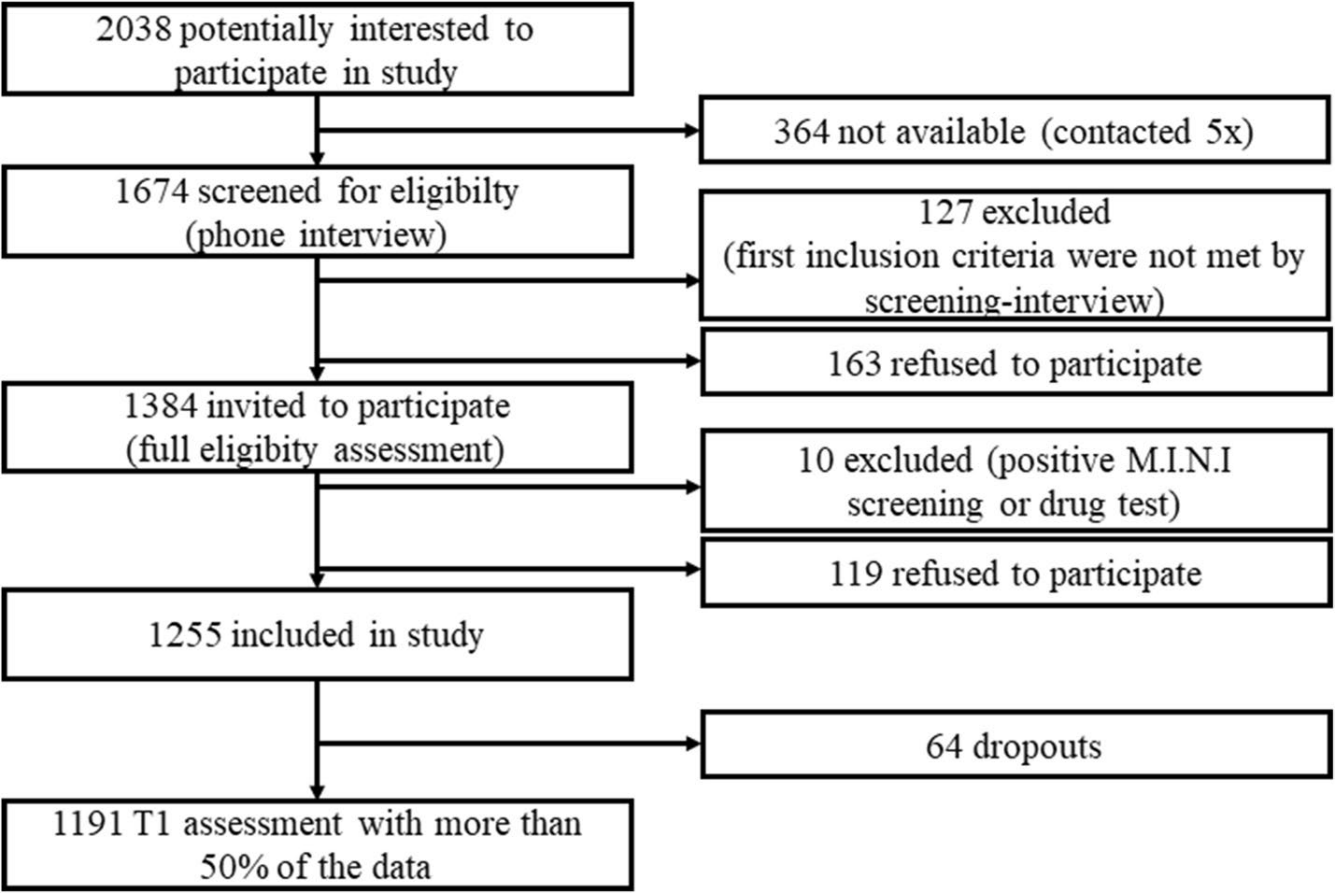
Flow chart of sample recruitment

Participants who met the screening criteria were invited for an initial briefing session and gave written informed consent. Furthermore, participants were assessed diagnostically on the International Neuropsychiatric Interview (M.I.N.I [16, 17]) to rule out the existence of any current mental disorders. For *n* = 10 participants, this screening was positive and further study participation was precluded (but referral to the outpatient departments of the respective participating study site’s psychiatric department for further diagnosis and treatment was offered). Figure 2 provides an overview of the recruitment process. If the diagnostic interview was negative, participants were eligible for study participation and enrolled for full study assessment. Additionally, participants who completed less than 50% of the first baseline assessment (B0/T0) were excluded from further study participation. For the on-site baseline assessments, participants are monetarily reimbursed with 60€. Participation at the online stressor monitoring is rewarded with a token system, were subjects can gain up to three tokens per monitoring and 12 tokens per year. Each token is worth 5€ and is exchanged against a monetary compensation.

### Procedures

According to the described study design, participants are invited on site to the baseline assessments for detailed characterization at 18-month intervals. In between baseline assessments, additional interim online stressor monitoring is applied every 3 months (see also Fig. 1). The detailed study design and applied assessment categories are described in detail below.

### Online database

Besides on-site assessments at baseline and additional interim online stressor assessments, participants fill out questionnaires in an online data base system (secuTrial© electronic data capture system, www.secutrial.com), which adheres to the Guidelines for Good Clinical Practice (GCP). The database provides an application for online assessments, through which the questionnaires assessing sociodemographic, lifestyle, and psychological variables are collected at the initial baseline assessment (B0/T0) and subsequent baseline assessments (B1/T6 and B2/T12), and the 3-month interim online stressor monitoring. All surveyed data are stored in the online data base system.

### Baseline assessments

Participants who fulfil the inclusion criteria are invited to the baseline assessments for detailed characterization at the respective study centers. These are conducted on 2 days, which are separated by no more than 7 days.

*B0/T0, day 1 (40–60 min)* After an initial briefing session, written informed consent is obtained and participants are registered in the study database, where a random individual identification number is being generated.

Afterwards, the International Neuropsychiatric Interview (M.I.N.I.) [16] is used to rule out the existence of any current mental disorders. Provided that all inclusion criteria are met, the actual baseline assessment begins with the conduction of the multifaceted empathy test (MET) [38]. Blood samples are taken from the non-fasting participants by venous puncture (2 × EDTA tubes; approximately 18 ml) to assess fluid biomarkers, including (epi-)genetic markers for genotyping. Blood samples are subsequently stored at refrigerator temperature until DNA isolation. Also, hair samples for cortisol determination are collected (see supplement for detailed information about biosample outcomes). For hair samples, two to three hair strains of at least 3-cm length are cut as close as possible to the scalp at the posterior vertex region [74]. Samples are wrapped in aluminium foil; the scalp end was marked and stored in a dark, dry place at room temperature until the end of complete baseline assessment. Given an average hair growth rate of 1 cm per month, earlier described by Wenning [75], the examination of 3-cm hair segment allows to assess cumulative hair cortisol concentration over a period of 3 months, consistent with assessed stressor load every 3 months. Hair samples are collected of all participants, who agree to submit a sample. Information about (chemical) hair treatments (i.e., colouring, perms, or using a strengthening iron) prior to sample collection is gathered. For participants with hair shorter than 3 cm, no hair samples are collected, but these participants will remain in the study. Since participants are asked to send in their hair samples by mail for the following five interim online stressor monitoring, they are also provided with packing material for the following five measurement time points (T1-T5; 3, 6, 9, 12, and 15 months). Anthropometric measurements (weight, height, hip, and waist circumferences) are conducted using a calibrated electronic scale (Seca, Birmingham, UK) with an accuracy of 0.1 kg for weight and a stadiometer (Seca) with an accuracy of 0.1 cm for height (participants were not wearing shoes). Waist circumference is measured to the nearest 0.1 cm midway between the lowest rib and the top of the iliac crest. Hip circumference measurement is taken around the widest portion of the buttocks. Both measures are conducted according to the WHO recommendations [76]. For the stool sample collection, the OMNIgene•Gut kits (DNA Genotek Inc. Ttawa, ON, Canada) are used. These consist of a tube of stabilisation liquid and a ball bearing. Two tubes, as well as a user manual for the stool sample collection at B0/T0 and the first 3-month measurement time point (T1), are handed out to the participants. They are asked to place stool faeces in the tube lid, which is designed to break up the faeces, and return the first tube at day 2, within 7 days. The second tube from T1 is returned by mail 3 months later. On arrival the samples are frozen immediately at − 80 °C.

Finally, participants are introduced to the online assessment application of the database for questionnaire assessments and asked to complete the questionnaires within a week. Questionnaires entail items assessing socio-demographics, mental health, life history, psychological, and lifestyle-related variables. The estimated completion time is 160 min. A complete list of applied questionnaires is given in Table 1a.

*B0/T0, day 2 (approx. 180 min)* First, participants hand in stool samples and take part in a drug screening. If the screening is negative, they are then asked to proceed. If screened positive, participants are excluded from the study. Then, participants are asked to fill out questionnaires measuring state-dependent variations in anxiety (i.e., State-Trait Anxiety Inventory; STAI-S [77, 78]) and the Positive and Negative Affect Schedule (PANAS; [50]). This is followed by a neuropsychological test battery, which assess the following potential neuropsychological resilience mechanisms: (a) cognitive flexibility, (b) emotional interference inhibition, (c) positivity bias, (d) volitional emotional regulation, and e) differential fear conditioning (discrimination) and extinction. Table 1b provides a description of the tasks and the measured potential underlying neuropsychological mechanisms. Subsequently, subjects are asked to participate in a detailed assessment of bodily composition and several physical fitness components. This part is optional for the participants (see supplements for details).

*B1/T6 and B2/T12, day 1 (approx. 40 min:* The procedure for these measurement time points substantially matches the one described for day 1 of B0. The International Neuropsychiatric Interview (M.I.N.I.) [16] is conducted again, to test for potential current mental disorders. If screened positively, subjects are not excluded from the study; however, trained staff decides in joint consultation with the participant, whether the participant is stable enough to complete the whole assessment. Nevertheless, referral to the outpatient departments of the psychiatric departments for further diagnosis and treatment will be offered for positively screened subjects. Participants are kept in the study to follow-up on the possible recovery from these mental illnesses and to investigate the skills, traits, and/or external factors (e.g., psychotherapy and hospital treatment) that might have helped them recover. Regarding biosamples, only one blood tube for epigenetic markers is taken and participants are provided with one tube for the stool sample at B1/T6 and packing material for hair samples for the following five measurement time points (3, 6, 9, 12, and 15 months).

*B1/T6 and B2/T12, day 2 (180 min)* The same procedure as described for day 2 of the baseline (B0/T0) is repeated.

Of note, in Frankfurt, the two described assessment days are conducted in 1 day, due to organizational reasons. Still, the questionnaires have to be filled out within 7 days. Furthermore, participants are provided with shipping material for the first stool sample at baseline.

### Interim online stressor monitoring (T1–T5, T7–T11)

In between the main assessments on-site, participants are asked to report their individual mental health status (GHQ-28) online, as well as individual exposures to life stressors every 3 months. Here, macro stressors, such as critical or major life events (CLE) and incidents of potentially traumatizing events (PTE), as well as microstressors, more precisely daily hassles (DH) are assessed, as described in detail in the procedure section and supplementary material. Additionally, participants send back hair samples (T1–T5, T7–T11) as well as gut samples (T1) via mail.

### Data management

Data collected via the online assessment application (SecuTrial database) (see above) are double-checked for consistency and plausibility. In case of missing, inconsistent or implausible data, participants are contacted by the study assistants. Furthermore, the SecuTrial database fulfils all requirements regarding data storage and protection according to national laws. Subproject-specific data (i.e., neuropsychological and physiological data) will be entered into sub-databases of the central Z03 database, designated to the specific subproject. Access to these sub-databases can only be granted by the leading principal investigators and very few assigned staff members and are only made available for the principal investigators of the specific subproject.

### Planned statistical analyses

The longitudinal procedures allow us to link individual properties, collected during baseline assessments on behavioral, biological, and neuropsychological levels, to stress reactivity in a longitudinal matter. By this, resilience processes can be identified. Resilience will be indexed by the reactivity of individuals’ mental health to stressors during 3-month time intervals in a ‘stressor reactivity’ (SR) score, derived using a residualization approach, previously introduced by Kalisch et al. [11], which investigates the relationship between stressors, operationalized by a combined score of daily hassles and life events over time, against general health, investigated using the GHQ score over time. The SR scores can then be calculated using a sliding window approach of overlapping time windows to reduce data loss, which reflects intra-individual temporal variability in resilience. We will investigate homeostatic adaptation, testing whether a potential resilience mechanism can satisfactorily predict SR over a longer period of time. Furthermore, we will investigate possible allostatic adaptations, which are hypothesized to take place when resilience mechanisms need to change or be adapted for a system to stay resilient, because the stressor exposure temporarily exceeds the system’s capacity. For the examination of allostatic adaption, changes in neuropsychological performance over time are assumed to be crucial. For more detailed information, we refer to Kalisch et al. [9, 11].

### Baseline data of recruited participants

For the resilience assessment, in total, a sample of 1255 healthy subjects from the Rheine-Main region spanning from Mainz (*n* = 624) to Frankfurt (*n* = 631) were enrolled, of which 1191 subjects completed at least 50% of the first baseline assessment (B0/T1) (see Fig. 2). Blood for DNA isolation has been acquired from *n* = 1,009 participants, stool samples for microbiome analyses from *n* = 1,041 participants, and hair for cortisol determination from *n* = 927 participants at baseline.

In the following, we describe the baseline data regarding outcome-based resilience measures and the assessment of perceived stress and stressors. Descriptive demographic statistics of the sample is shown in Table 2. Participants’ age ranged from 18 to 50 years and the sample is biased towards females (66%). Although 8% of the sample participants are of non-German nationality, all of them have sufficient knowledge of the German language, as is inferred from the screening interview and the interactions during the assessment. A considerably high number of participants are non-married (80%), most likely due to their rather young age. The overall education level can be considered as being rather high, since nearly half of the sample (44.5%) holds a university degree. More than half (53.4%) of the B0/T0 sample participants are still obtaining a degree at the time of the B0/T0 measurement.

**Table 2:** Baseline data of the LORA study sample (*N* = 1191)

Variable	*n*	Percentage	Range
*Gender*			
Female	783	65.9	
Male	406	34.1	
*Age (M/SD) total*	1188	28.59 (7.96)	18–50
< 20 years	63	5.30	
20–29 years	724	60.94	
30–39 years	246	20.71	
> 40 years	155	13.05	
*Nationality*			
German	1083	91.86	
Other European countries	51	4.32	
Others (%)	45	3.82	
*Marital status*			
Non-married	867	80.50	
Married	182	16.90	
Separated	9	0.84	
Divorced	16	1.49	
Widowed	3	0.28	
*Highest educational achievement*			
No school-leaving qualification	1	0.09	
School-leaving certificate	2	0.19	
Certificate of Secondary Education	29	2.69	
School-leaving examination	420	38.92	
Completed vocational training	147	13.62	
University degree	480	44.49	
*Employment*			
Full time	342	31.78	
Part time	131	12.17	
Part time due to health issues	3	0.28	
No employment due to reasons other than health issues	24	2.22	
No employment due to health issues	2	0.19	
Currently obtaining an education	574	53.35	
Number of previous life events (lifetime), *M*(SD)	1188	11.81 (7.14)	0–39
Number of daily hassles (past 7 days), *M*(SD)	1149	63.66 (27.14)	0–175
GHQ, overall, *M*(SD)	1183	16.55 (7.62)	0–49
BRS score, *M*(SD)	1182	3.76 (0.67)	1–5
PSS score, *M*(SD)	1186	12.46 (5.74)	0–31

Notes: Percentage based on valid data; mean and standard deviation based on all obtained data, extreme outliers excluded

**Fig. 3:**
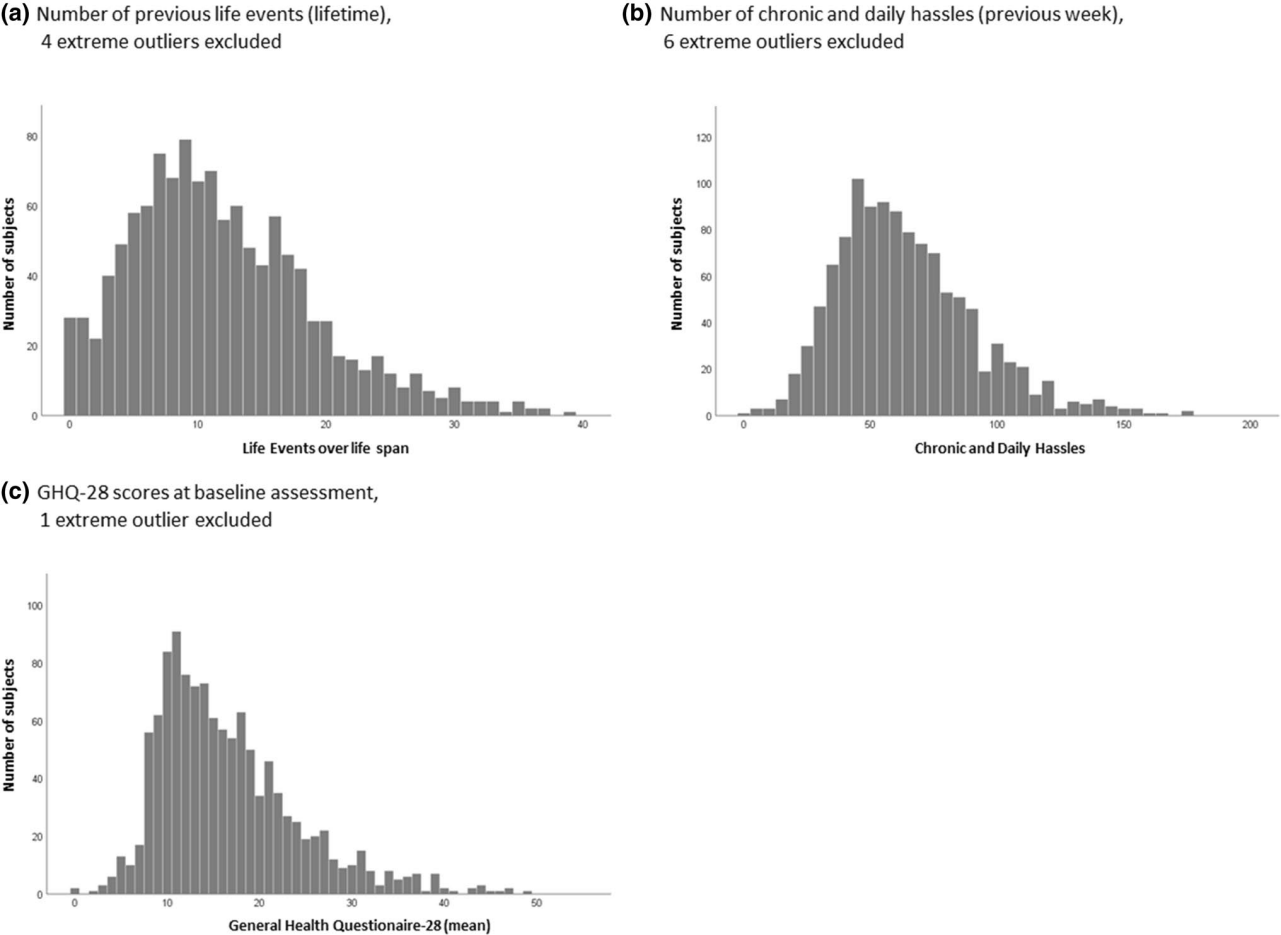
Frequency of previous life events, chronic and daily hassles, and mental health

Based on all data from the baseline assessment (see Supplement for detailed description of the scoring), excluding extreme outliers (± 4 or more standard deviations of the mean), participants reported a mean of 11 major life events during lifetime (Fig. 3a). Furthermore, the number of experienced life events correlated positively with participant’s age (*r* = 0.36, *p* < 0.001). The median number of chronic and daily hassles was 60, with a mean = 63.66 (Fig. 3b). On average, reported daily hassles equal almost ten hassles per day. In the self-assessment test of perceived stress (PSS-10), most participants indicated a stress level of 12 (median = 12), which can be considered as rather low; 33.6% report to be moderately-to-highly stressed.

Reported psychological symptoms at baseline, assessed by the GHQ-28, were non-normally distributed, with positive skewedness of 1.49 (SE = 0.07) and kurtosis of 1.45 (SE = 0.14). As shown in Fig. 3c, 993 participants reported symptoms below the threshold of 23/24 points [19]. Of note, the 190 (14%) subjects scoring above the diagnostic threshold did not fulfil the criteria of a mental disorder according to the initial M.I.N.I. assessment.

### Current and future work

As of July 2019, the first baseline assessment (B0/T0) has been completed for all participants at both sites. Interim online stressor monitoring, as well as the second baseline measurement (B1/T6) are ongoing. The third baseline measurement (B2/T12), which is 3 years post-study onset, will be assessed in the first subjects in Mainz and Frankfurt in February 2020. The unique study design and analysis scheme, as outlined in Fig. 1 will be continued. We plan to follow up the 1191 participants enrolled at B0/T0 (baseline) for another 3 years (up to early B4/T24).

### Discussion, limitations, and outlook

We have postulated that resilience is not simply the absence of mental health problems, but rather a process that can be operationalized as the amount of stress and daily hassles a person encounters over time in relationship to the general health outcome that person shows [2].This calls for the use of longitudinal approaches to investigate resilience mechanisms in more depth. The LORA study uses a unique design to assess resilience over a time period of at least 3 years, being able to observe resilience mechanisms, while they occur in response to modern-life stressors. This is done by extensively capturing participant’s stressor load with intermediate online stressor monitoring every three months, representing a very high sampling rate. This monitoring not only assesses the influence of life events, but also captures modern every-day life stressors, the so-called daily hassles, assessed with the Mainz Inventory of Microstressors (MIMIS; [25]), giving a good overview of minor and major stressors, together with their temporal extend. The stressor monitoring includes also hair cortisol samples, resulting in a more objective way to capture undergone stress and activation of the hypothalamic–pituitary–adrenal (HPA) axis over the last 3 months [79].

Moreover, several cognitive abilities, which are assumed to be affected under stress, are tested with a neuropsychological test battery during the baseline assessment time points every 18 months. The repeated application of this neuropsychological test battery in the LORA study will give new insights into the duration of these influences, possible interindividual differences, and are a proxy for the underlying neurobiological processes taking place in resilience. Most importantly, this neuropsychological battery may shed light on the resilience mechanisms taking place in between assessment time points.

Resilience mechanisms might also include alterations of gene expression, e.g., via epigenetic modifications such as DNA methylation. Thereby the environment may interact with gene regulatory networks, such as the glucocorticoid system. Studies in rats and postmortem studies in humans have, e.g., shown that an increase in the glucocorticoid receptor (encoded by *NR3C1*) promoter methylation later leads to higher stress resilience, pointing to an allostatic adaption of the stress axis as a consequence of life events [80]. These and other studies provide first clues on how the genetic makeup might interact with the environment to affect resilience outcomes. Furthermore, there might be genetic variants that could more directly influence adaptation processes to stressors [81]. To this end, DNA is sampled in LORA to generate genome-wide SNP analyses and epigenome-wide analyses to further examine their link with resilience mechanisms.

In the LORA study, also data concerning physical fitness levels and body composition are collected, since there is plenty of evidence that physical fitness, achieved through regular physical exercise, yields physical and mental health benefits by influencing stress responses [82, 83]. Furthermore, Mujica-Parodi and colleagues [84] showed that increased body fat was related to elevated cortisol reactivity and decreased cognitive performance, particularly spatial processing, selective attention, and working memory. As such, body fat percentage can also have an impact on stress responsivity and cognitive performance, rendering it a worthwhile resilience mechanism to investigate.

As is known from recent studies, the gut–brain axis also plays an important role in stress responses. For example, stress was shown to reduce the bacterial levels in the gut flora of a student sample during exam weeks, indicating an influence of stressor exposure on the gastrointestinal microflora [85]. Hence, the stool samples collected in the present study can give important information on the influences of stress on a person’s microbiome and how a certain microbiota composition determines stress responses in the individual.

A total of 1255 participants have been recruited in the LORA study, which exceeds the originally planned 1200 participants. Of those enrolled participants, 1191 participants have completed more than 50% of the assessment at baseline. Slightly more women are enrolled in the study (65.9%) and most participants are rather highly educated, with 44.9% having a university degree and more than half of the sample currently obtaining one. However, highly educated samples are a phenomenon commonly observed in large-scale studies, such as the present one [86]. University students are a quite homogenous group of people. They are easy to recruit and potentially have a higher economic motivation to participate in studies and also have sufficient time to take part in longitudinal studies, like the present one. However, they might not be representative for the world population [87]. Therefore, the results should be interpreted with caution. It is unclear whether students are a group of people who are usually exposed to relatively small amounts of stress, or even to a lot of stress and if they have better coping mechanisms compared to the world population. However, the rather low amount of perceived stress on the PSS-10 in the present study infers a rather reduced level of stress exposure in the current sample compared to other studies (e.g., [88]). Participants show a medium amount of previous life events, with a mean = 11.81, and a rather low number of daily hassles with on average almost 64 hassles (mean = 63.66). Participants’ general health was good, as indicated by a GHQ mean score of 16.55, with none of the participants showing any psychiatric disorders on axis I, as assessed on the M.I.N.I. interview [16]. The mean BRS score was within the range of previously reported BRS means in comparable German populations (*M* = 3.58 and *M* = 3.37; [31] and *M* = 3.35; [90]). Hence, the self-reported ability to bounce back and recover from stress in the current sample was comparable to previous observations.

Several possible limitations of the project have to be considered. First, as with all longitudinal projects, there is always some amount of drop-out over time, especially for studies running over several years such as the present one. However, in the presented project, the drop-out rate is rather low with 15.3% from the first baseline to the second baseline assessment and an overall drop-out rate of 21% over all time points that are currently assessed, undershooting the expected overall drop-out rate of 25%. Second, due to the vast amount of data collected in different modalities, the project places a rather high burden on the participants. As such, sampling biases, such as a self-selection bias, can be expected as well as missing or incomplete data, especially during the interim online stressor monitoring. Not all participants are available over such a long time period or able to come to the baseline measurements during the week. This might result in a rather young sample, consisting of participants who are not working full time yet. Although the present study sample consists of a rather high amount of young and highly educated participants, there is still a good variance in the sample, assuring a generalizability of the findings to the general population. Also, such small sampling biases are rather often in longitudinal studies. Drop-outs and missing data are reduced by a repeated reminder scheme for online stressor monitorings via e-mail, regular booster mails to participants, thanking them for their study participation, give-aways with the LORA logo printed on them. Furthermore, participants will be notified of publications of resilience research to become aware of the impact of their contribution to science.

In sum, the LORA study is a unique starting point for a more detailed investigation into resilience mechanisms, in that it investigates these mechanisms, while they actually occur, rather than in a retrospective fashion. As such, the full process, from the occurrence of stressors to a possible recovery from them, can be observed online. In addition, by investigating healthy participants over time, the presented project applies a new and more fine-grained approach to investigate resilience. While the previous studies have mainly focused on resilience by assessing participant’s health outcomes (i.e., stress-related psychiatric disorders vs. no disorder), linking them to stressors retrospectively, the presented study uses a forward approach by observing stressors and directly assessing their influence on the human organism. This is done by investigating many distinct domains with respect to their link with resilience and using multiple techniques, such as biological samples, neuropsychological performance, self-rating questionnaires, diagnostic interviews, and individual fitness. Therefore, the LORA study will be able to give information on how these different domains interact and influence each other with regard to resilience outcomes. Furthermore, through the application of more objective measures, such as biological samples, the presented project can advance resilience research. In sum, the LORA study uses a promising approach to shed light on the mechanisms applied in the resilience process.

## Electronic supplementary material

Below is the link to the electronic supplementary material.

Supplementary file1 (DOCX 30 kb)
