# Supplementary material for: Longitudinal determination of resilience in humans to identify mechanisms of resilience to modern-life stressors: the longitudinal resilience assessment (LORA) study
Source: Eur Arch Psychiatry Clin Neurosci. 2020 Jul 18;271(6):1035–51. doi: 10.1007/s00406-020-01159-2 (PMC8354914; doi:10.1007/s00406-020-01159-2)
Supplement: Supplementary file 1 — Supplementary file1 (DOCX 30 kb) [file 406_2020_1159_MOESM1_ESM.docx]

**Supplement**

Longitudinal determination of resilience in humans to identify mechanisms of resilience to modern-life stressors: The longitudinal resilience assessment (LORA) study

submitted to European Archives of Psychiatry and Clinical Neuroscienes

Authors: Chmitorz, A. Neumann, R. J., Kollmann, B., Ahrens, K. F., Öhlschläger, S., Goldbach, N., Weichert, D., Lutz, B., Plichta, M.M., Fiebach, C., Wessa, M., Kalisch, R., Tüscher, O., Lieb, K., Reif, A.

Corresponding author: B. Kollmann

Bianca Kollmann, Leibniz Institute for Resilience Research gGmbH, Wallstraße 7, 55122 Mainz, Germany, email: bianca.kollmann@lir-mainz.de

**Detailed description of the scoring of the most relevant measuring instruments for baseline and the interim online stressor monitoring assessments:**

In the assessment of the stressor load, critical life events and daily hassles are treated separately, in order to highlight that they feature distinguishable sources of stress [2]. Regarding macro stressors, exposures to critical or major life events prior to study begin were collected retrospectively, using an adapted German version of a standard life events (LE) checklist from Canli and colleagues (Life Experiences Questionnaire [21]). The questionnaire lists critical or major life events and incidents of potentially traumatizing events (i.e. job loss, divorce, death of a loved one) subsumed under ‘critical life events’ (CLE). The adapted German version contains 27 items, for which subjects indicated whether this event occurred in their lifetime and at what time point. At the first baseline assessment up to five separate time points (year) could be indicated per event. The affective impact per event is also indicated, ranging from 0 (not at all affected) to 4 (very much affected). Events, also those that occurred more than once, are counted by summing up the number of occurrences, independent of their valence. For all subsequent assessments of life events, participants are asked to report the occurrence of life events within the last three months. With regard to micro stressors, more precisely chronic stressors and daily hassles (DH) are assessed using the Mainz Inventory of Microstressors (MIMIS), which was recently developed and validated by our group [22, 24]. Examples of MIMIS items are commuting, arguments with colleges at work, work overtime or noise. Here, participants are asked to retrospectively report the number of days the stressors occurred from a list of 58 DH (ranging from 1-7 days) and the mean stressfulness of these events within the past seven days, including the day of assessment. Ratings of stressfulness are made on a five-point Likert-scale, ranging from 1 (not at all affected) to 5 (very much affected). For analysis, DH were calculated by multiplying the total amount of all hassles reported in the past week with the reported number of days on which a hassle occurred (range: 0 – 58*7 days = 406) with no weighting by severity. For the assessment of general health status, the German version of the General Health Questionaire-28 (GHQ-28; [87, 18]) is used. The GHQ-28 is a 28-item measure of emotional distress and rates participant’s subjectively reported health over the last couple of weeks on a four-point Likert scale ranging from 0 (least symptomatic answer) to 3 (most symptomatic answer) with the possible total score ranging from 0 to 84 [18]. Items are assessed on four scales, i.e. somatic symptoms, anxiety/sleeplessness, social dysfunction, and severe depressive symptoms. The threshold for distress is a total sum score of 23/24. Subjectively perceived stress was assessed by a German version of the Perceived Stress Scale (PSS; [27]). The German version is a translation of Cohen’s PSS entailing 10 items (translated by Büssing, A.). The scale consists of ten items and assesses how much participants were negatively affected by stress over the past month. Answers are assessed on a five-point Likert scale ranging from 0 (“never”) to 4 (“very often”). Items 4, 5, 7, and 8 are inverted. A sum score is calculated over all items (range: 0-40). Higher values indicate more perceived stress. All three described tests are applied at all baseline assessments in an 18 month interval, as well as every three months at the interim online stressor monitoring. The German version of the Brief Resilience Score (BRS; [30, 32]) assesses participants’ likeliness to agree with six different remarks about how they cope with stressful incidences. Responses are rated on a five-point Likert scale, ranging from 1 (“totally do not agree”) to 5 (“fully agree”). Items 2, 4, and 6 are inverted items, so that they are scored reversely. Total scores are obtained by summing up the scores of all six items and calculating a mean score [31]. Higher values indicate a higher ability to recover from stress.

**Biosample outcomes**

*Blood samples*: Whole blood samples are taken for genotyping. We aim to identify genetic markers with genome wide SNP analysis (GWAS), which allows us to test hypothesis-free, unbiased analyses of biological specimens. Furthermore, epigenetic analyses of leukocyte DNA are of special interest to assess the effects of stress on the epigenome and its relation to resilience. Therefore, polygenetic risk scores and genome-wide methylation patterns will be used to identify variations in methylation (i.e. dopamine signal transduction, serotonin or neurotrophin transduction).

*Stool samples*: For gut microbiome sequencing marker sequence 16s ribosomal RNA (rRNA) gene will be used. This approach allows for the exploration of bacterial phylogeny, taxonomy, or functional profiling, as this genetic marker contains conserved hypervariable regions [87]. Once the bacteria composition has been identified, associations between bacteria and measured parameters, here particularly stress exposure and resilience mechanisms, can be explored.

*Hair samples*: Being incorporated into the growing hair, hair cortisol concentrations (HCC) are assumed to provide a valid index of retrospective systemic reflection of integrated long-term secretion of the glucocorticoid cortisol over periods of several months [73. We aim to utilize HCC to answer the question of its mediating role in chronic stress and health related conditions.

**Additional assessment of bodily composition and physical fitness at baseline assessments**

Subsequently to the neuropsychological test battery at baseline assessments (B0-B2), subjects are asked to participate in a detailed assessment of bodily composition and several physical fitness components. Assessments include the measurement of skinfold thickness, using a caliper (holtain skinfold calipers) to perform a seven-site skinfold body fat measurement according to standard procedures recommended by the International Society for the Advancement of Kinanthropometry (ISAK; [88]). Cardiorespiratory fitness (CRF) is measured by the Chester Step Test in order to predict maximal oxygen uptake (VO2max) [89]. Maximum muscular strength is assessed by a hand grip strength analogue dynamometer (Takei Scientific Instruments Co. Ltd.; [90]) and a standing broad jump test to measure lower limb explosive strength [91].
